# Embolisation of Type 2 Endoleaks Associated with Sac Expansion—Outcomes from a Single-Centre Cohort

**DOI:** 10.1007/s00270-025-04272-8

**Published:** 2026-01-29

**Authors:** Joo-Young Chun, Shyamal Patel, Seyed Ameli-Renani, Vyzantios Pavlidis, Robert Morgan

**Affiliations:** St Georges Hospital NHS Foundation Trust, Blackshaw Road, Tooting, London, England

**Keywords:** AAA, EVAR, Endoleak, Embolisation, Sac growth

## Abstract

**Purpose:**

To describe the outcomes of a patient cohort following transcatheter embolisation for type 2 endoleaks associated with sac expansion.

**Materials and Methods:**

A retrospective single-centre observational study was performed between September 2005 and June 2023. Technical success rates and long-term outcomes were evaluated including technical factors associated with endoleak recurrence and rupture. One hundred transcatheter embolisations were performed for type 2 endoleaks in 72 patients (64 male and 8 female).

**Results:**

Technical success (cessation of flow in the endoleak on angiography) was achieved in 77/100 (77%) procedures. Clinical success (freedom from sac growth on surveillance) was achieved in 82% at 12 months, 70% at 24 months and 59% at 60 months. Persistent endoleaks were found in 27%, with 7% developing a new type 2 and 14% developing new type 1, 3 or 5 endoleaks. The rupture rate was 7%, including 2/7 persistent type 2 endoleaks, 4/7 new type 1 or 3 endoleaks and 1/7 type 5 endoleak. Embolisation was performed either via a transarterial route (74%) or via direct sac puncture (24%), the latter demonstrating a significant correlation with technical success (*p*=0.018).

**Conclusions:**

This study confirms the importance of embolisation as the main treatment modality of type 2 endoleaks with freedom from sac growth achieved in 70% of patients at 24 months. However, this remains a complex entity with persistent sac growth, risking the development of type 1 or 3 endoleaks, which carry a risk of late sac rupture.

**Graphical Abstract:**

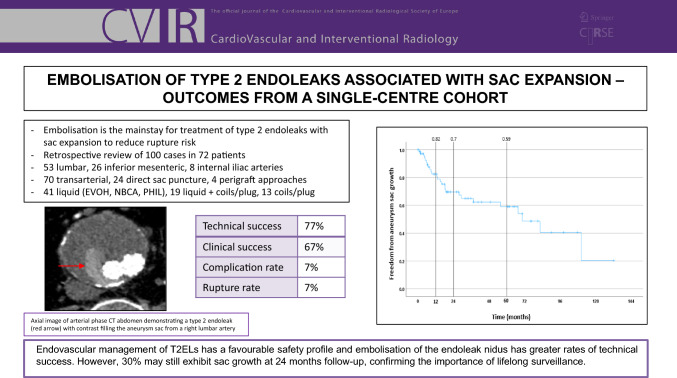

## Introduction

Type 2 endoleaks (T2EL) are the most common subtype, occurring in up to 20–30% of patients after endovascular aneurysm repair (EVAR). T2ELs represent retrograde filling of the aneurysm sac from aortic or iliac branches, most commonly the inferior mesenteric (IMA) and lumbar arteries (LA)[1–3]. T2ELs are low-flow and often transient, resolving spontaneously in up to 50% [4, 5]. Isolated T2ELs have been associated with a low risk of aneurysm sac rupture of <1% [6]. Some authors have demonstrated no survival benefit following treatment of T2EL compared to conservative management [7–9]. Conversely, others have identified T2EL to be an independent risk factor for sac expansion, re-intervention, and late aneurysm sac rupture [10–12]. The current consensus is to consider intervention in persistent T2EL when associated with significant sac expansion, typically >10mm since the first post-treatment scan [1, 2, 4, 13–16].

The mainstay of intervention is embolisation of the feeding arteries and endoleak cavity [1, 2, 16]. The technique depends on EL anatomy and operator experience; challenging endoleaks may require multiple approaches. The main access routes are transarterial and percutaneous direct sac puncture (DSP) [1, 16–18].

Various embolic agents may be used including metallic coils and liquid embolic agents (including Ethylene vinyl alcohol copolymer [EVOH] and n-butyl cyanoacrylate [NBCA]), with no good-quality evidence available to decide which agent is optimal [1, 16].

The aim of this study was to assess the outcomes of T2EL embolisation in a single-centre cohort.

## Methods

A retrospective observational study was performed at our tertiary referral centre between September 2005 and June 2023. The inclusion criteria were patients with persistent T2EL associated with aneurysm sac expansion (>10mm in maximal diameter over 12 months) who were recommended for intervention following discussion in the vascular multidisciplinary meeting. A single patient with retrograde filling of the aneurysm sac due to a malpositioned fenestrated graft covering the celiac axis was also included. Written informed consent was obtained from each patient. Local Ethics Committee approval for retrospective studies such as the current study is not required at our institution.

### Technique

All procedures were performed under conscious intravenous sedation and local anaesthesia.

**Fig. 1:**
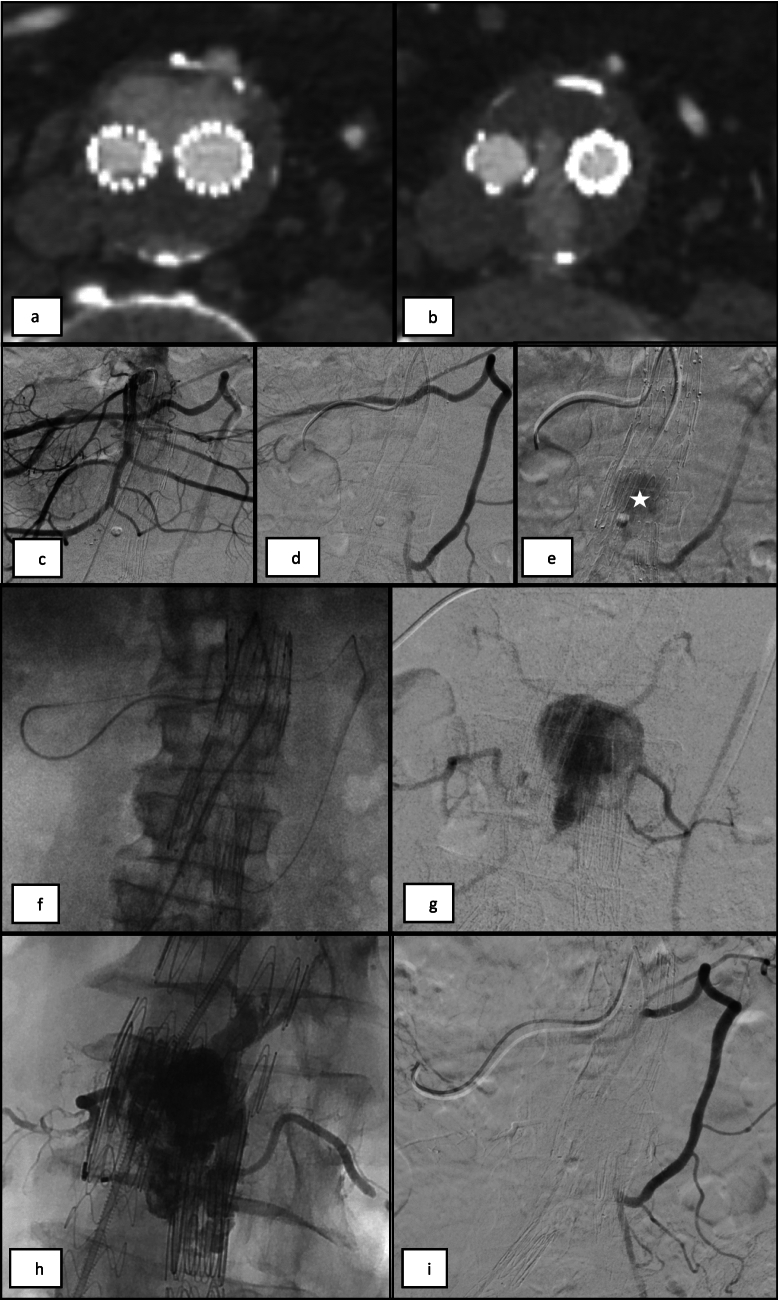
Transarterial embolisation of an IMA T2EL. (a, b) CTA imaging demonstrates a T2EL arising from the IMA with contrast filling the aneurysm sac anteriorly and extending between the iliac limb endografts. (c-e) Selective angiogram from the middle colic artery of the SMA opacifies hypertrophied arc of Riolan. IMA and endoleak nidus within the aneurysm sac (*). (f) A 2.3Fr microcatheter was advanced through the arc of Riolan and IMA origin, into the aneurysm sac. (g) Angiogram from the aneurysm sac opacifies the EL nidus and multiple lumbar outflow branches. (h) Embolisation of T2EL with NBCA injected via the microcatheter spreads into the sac, lumbar vessels and IMA. (i) Completion angiogram demonstrates no further endoleak

**Fig. 2:**
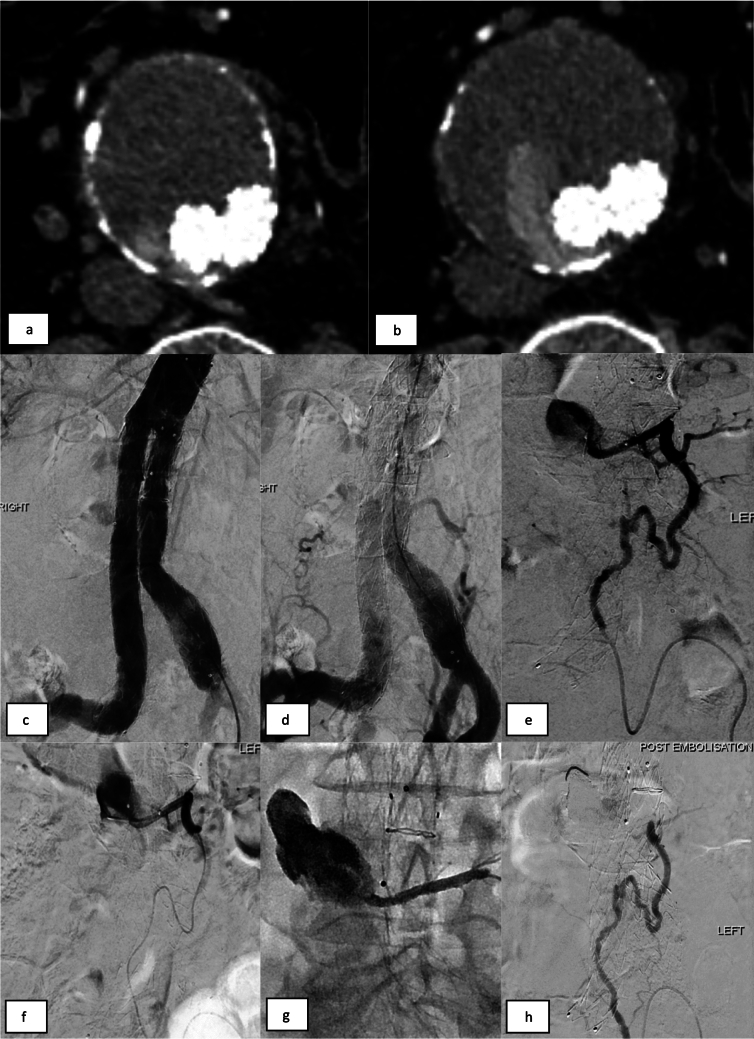
Transarterial embolisation of a left lumbar artery T2EL. (a, b) CTA imaging demonstrates a T2EL arising from a left lumbar branch with contrast filling the aneurysm sac posteriorly and around the right iliac limb endograft. (c-e) Catheter angiograms from the aorta and left iliolumbar artery demonstrate retrograde filling of the aneurysm sac via the left lumbar branch. (f) A 2.7Fr microcatheter was advanced into the aneurysm sac and an angiogram confirms catheter tip position and excludes communication with any spinal branches. (g) 9 ml of EVOH (Onyx 34L) injected via the microcatheter spreads into the sac and inflow lumbar vessel. (h) Completion angiogram confirms no further endoleak

The transarterial approach [1, 16] was usually performed first, during the same procedure as the diagnostic angiogram. The common femoral artery was the standard access route and a variety of angled and reverse-curved 5Fr catheters were used to select target arteries. The endoleak was approached via collateral pathways—the arc of Riolan for IMA T2ELs or via the ipsilateral iliolumbar artery for LA T2ELs (Figures 1, 2). A microcatheter (2.0–2.7Fr) was advanced into the aneurysm sac and endoleak cavity for embolisation. The transiliac perigraft approach was utilised if feasible and the transarterial route failed [1, 16].

**Fig. 3:**
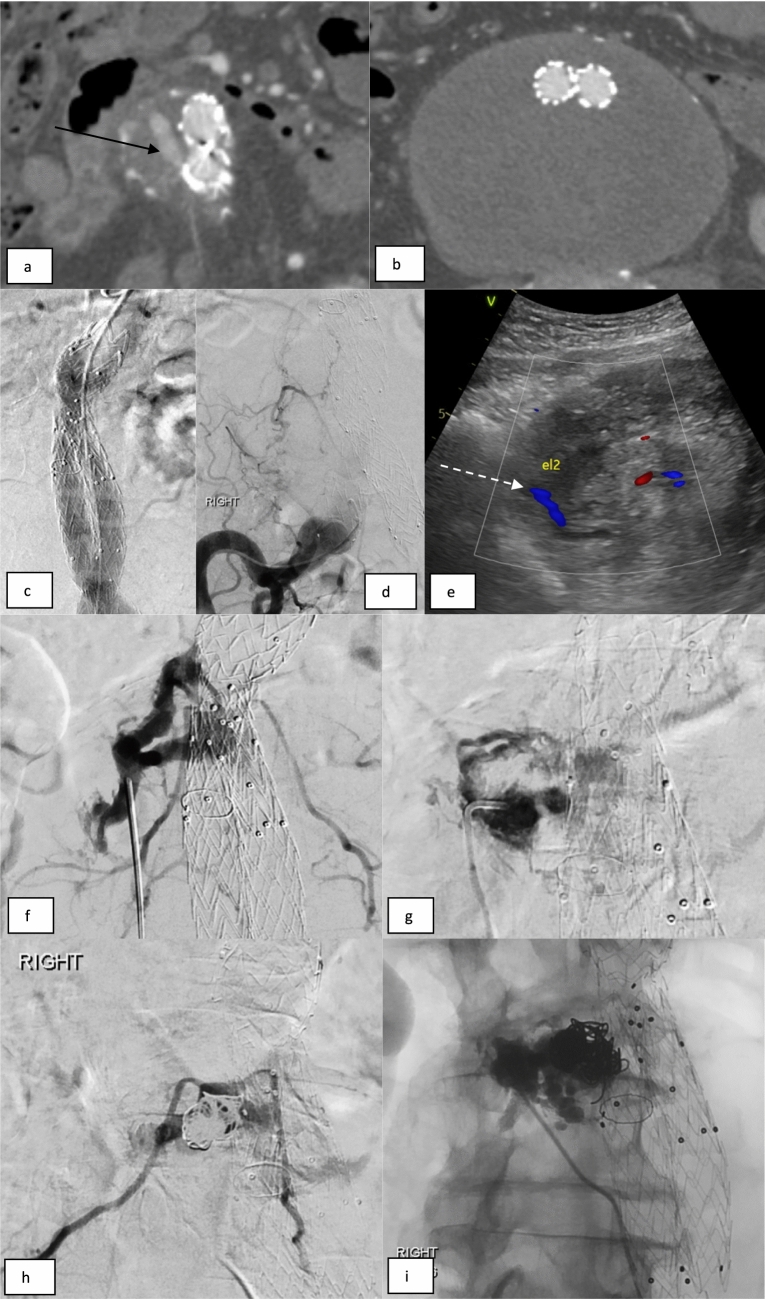
Direct sac puncture with embolisation of bilateral lumbar T2ELs. (a, b) CTA imaging demonstrates an endoleak of unclear source (arrow). (c, d) Catheter angiograms from the aorta and right common iliac artery do not show a clear endoleak. (e) A duplex ultrasound demonstrates a T2EL endoleak in the aneurysm sac (dashed white arrow) and confirms a safe percutaneous transabdominal window for access. (f) Access was obtained under direct US guidance with an 18G trocar needle. Angiogram from the aneurysm nidus demonstrates contrast opacification of the aneurysm sac and bilateral lumbar ‘outflow’ branches. (g) An angiogram from a 4Fr sheath and catheter sited in the aneurysm sac. (h) Several pushable coils were placed within the aneurysm sac prior to liquid embolisation. (i) 4mls of NBCA injected via the catheter spreads into the sac and out into the lumbar vessels bilaterally

DSP was often reserved for cases where the transarterial route had failed or was felt unlikely to be successful. This was usually performed on a different date than the transarterial attempt and from either an anterior or posterior route. Posterior access was performed using fluoroscopic/CT guidance with the patient prone. Transabdominal DSP was performed in the supine position under ultrasound guidance Figure 3. An 18-20G trocar needle was navigated into the EL nidus. Embolisation could be performed directly via the outer cannula. Alternatively, the cannula was exchanged for a 4Fr sheath, allowing a catheter/microcatheter to be manipulated into the inflow/outflow vessels as required.

The transcaval approach was attempted if above techniques failed or were unsuitable. This utilized an angled sheathed catheter and needle such as in a transjugular intravenous porto-systemic shunt (TIPS) set to puncture through the IVC and obtain access to the endoleak cavity [1, 16].

### Embolic Agents

Many embolic agents have been used for the embolisation of T2ELs, either alone or in conjunction: NBCA (Glubran 2–GEM srl, Turin, Italy), EVOH (Onyx–EV3, Medtronic, Santa-Rosa, CA), PHIL (Precipitating Hydrophobic Injectable Liquid – Microvention, Terumo, Aliso Viejo, CA), pushable and detachable macro/microcoils, vascular plugs, particles (ranging from 700–1100-micron calibrated spheres or 500–710-micron non-spherical polyvinyl alcohol [PVA] particles). The selection of the specific embolic agent in each case was based on microcatheter position, endoleak morphology and operator preference. NBCA was diluted with lipiodol in a 1:3–1:4 ratio. EVOH was used in the Onyx 34/34L formulation for higher viscosity.

### Outcome Measures & Statistical Analysis

The primary outcome measures were persistent aneurysm sac growth, re-intervention, and late aneurysm rupture. Technical success was defined as cessation of flow in the T2EL on completion angiography. Clinical success was defined as freedom from sac growth (>5 mm increase in maximal diameter of the aneurysm sac compared to the pre-embolisation dimensions) on surveillance imaging. Technical factors such as the approach or embolic agent were analysed to identify differences in clinical success with univariate Chi-squared (*X*^2^) analyses. Odds ratios (OR) with 95% confidence intervals were also calculated (Haldane-Anscombe correction and Baptista Pike method). Freedom from re-intervention, sac growth and aneurysm rupture were estimated using the Kaplan–Meier method (SPSS v29, IBM).

### Follow-up

All patients underwent surveillance duplex ultrasound (US) or CT angiography (CTA) in the arterial phase. Unenhanced and venous phases were reserved for problem solving and are not part of the routine follow-up protocol. Follow-up imaging was reviewed for the presence or absence of the treated T2EL, any new EL, aneurysm sac measurements, and aneurysm rupture.

## Results

### Demographics

Seventy-two patients underwent 100 embolisations for T2EL. There were 64 men and 8 women with a mean age of 74.5 years. The mean follow-up was 28.6 months. The majority of patients had undergone standard bifurcated EVAR (80%), fenestrated EVAR in 10% and in combination with iliac branch device in another 10%. The most frequently used stent grafts were Zenith (Cook, Bloomington, IN), Talent (Medtronic, Minneapolis, MN) and Endurant (Medtronic). The mean AP aneurysm sac size at the time of EVAR was 60.5 mm (+/-12.5 mm) compared with 72.9 mm (+/-15.8 mm) at the time of first embolisation. The time to embolisation from the initial EVAR was variable with a mean of 43.6 months, ranging from 0.5-171.5 months. Forty-nine patients (68%) underwent one embolisation procedure, with 23 patients (32%) requiring more than one procedure. Repeat procedures were performed either due to clinical failure following embolisation or technical failure of one approach and a subsequent re-attempt using an alternative approach. The most frequent source of T2EL was the LAs followed by the IMA (Table 1). In 12/100 cases, there was more than one target artery which required cannulation and embolisation, for example a LA and an accessory renal artery.

**Table 1:** Target artery for T2EL embolisation

	Number (%)
Target vessel	
Lumbar artery	53 (53)
Inferior mesenteric artery	26 (26)
Internal iliac artery	8 (8)
>1 target artery	12 (12)
Celiac trunk	1 (1)

### Outcomes

Technical success was achieved in 77/100 procedures. The reason for technical failure (23/100) was the inability to reach the target artery or aneurysm sac for safe or adequate embolisation. This was encountered most frequently when LAs were the target vessel (17/23). Of the 23 technical failures, 18 were attempted transarterial, 2 transiliac perigraft, 1 DSP, 1 transcaval and 1 direct gluteal artery puncture. Thirteen of these patients re-attended for a second attempt with DSP and went on to have a technically successful embolisation.

Clinical success was achieved in 67 embolisations (67%). This did not correlate directly with the endoleak status on post-procedure imaging—only 44% were endoleak-free with the remaining cohort demonstrating either persistent T2EL or a new EL (Table 2). More than half of the cohort with persistent T2EL did not require re-intervention as the sac diameter remained stable. Conversely, 12 patients developed a new type 1 (T1) or type 3 (T3) EL requiring additional treatment. Eight of these patients underwent endovascular management, including aortic cuff, stent graft relining and limb extension.

**Table 2:** Endoleak status on post-procedure imaging

Endoleak status	Number (%)
No endoleak	44 (44)
Persistent/Partially treated T2EL	35 (35)
Technical success of T2EL embolisation	20/35 (57)
Stable/Reduced sac size	18/35 (51)
Increased sac size	17/35 (49)
Repeat embolisation performed	6/35 (17)
Rupture (without evidence of T1/T3EL on imaging)	1/35 (3)
New T2EL	7 (7)
New T1 or T3 (with or without T2EL)	12 (12)
New T5EL	2 (2)

**Fig. 4:**
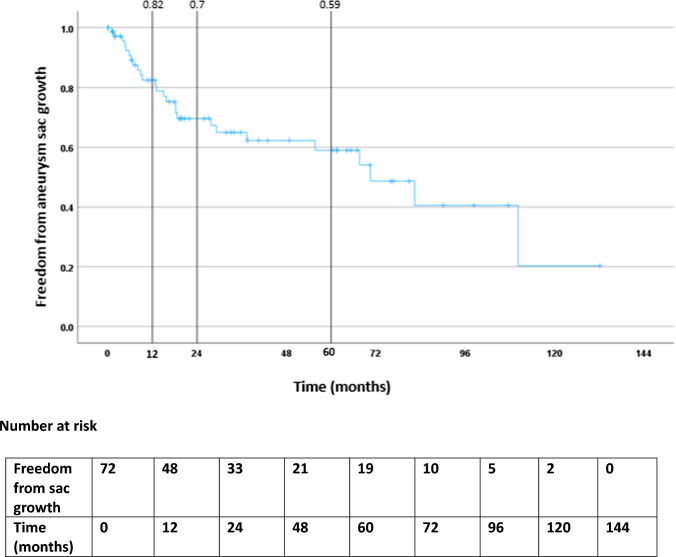
Freedom from aneurysm sac growth following embolisation of T2EL in the cohort over the follow-up period

**Fig. 5:**
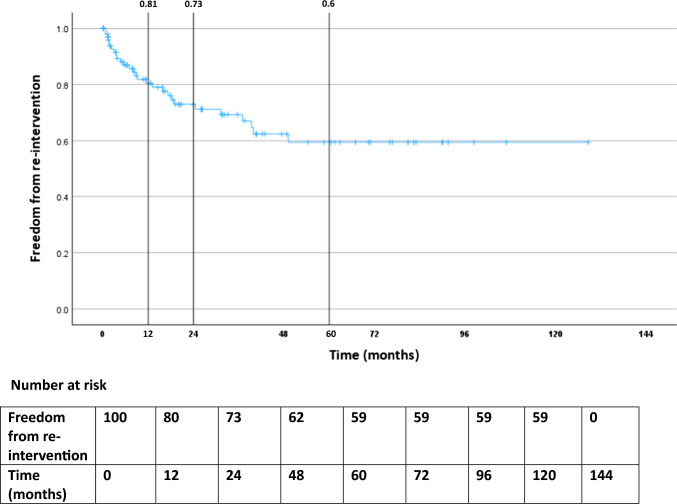
Freedom from repeat T2EL embolisation in the cohort over the follow-up period

The cumulative freedom from sac expansion was 82% at 1 year, 70% at 2 years and 59% at 5 years (Figure 4). Freedom from repeat embolisation was 81% at 1 year, 73% at 2 years and 60% at 5 years (Figure 5). More than half of the patients who underwent repeat procedures (13/22 – 63.6%) had had a technical failure from a transarterial approach and were brought back for a second attempt using the DSP technique. Three patients underwent surgical management of persistent T2ELs after technical failure to embolise—2 laparoscopic clipping of the IMA and 1 surgical ligation of a common iliac artery aneurysm.

There were 7 confirmed cases of aneurysm rupture. Four of these were associated with a new T1 or T3 endoleak following successful T2EL embolisation and no residual T2EL on follow-up imaging. Of the remaining 3, one had undergone a technically unsuccessful embolisation, one developed recurrent T2EL following a successful embolisation, and another had unexplained sac expansion following multiple embolisation procedures. The mean interval from initial embolisation to aneurysm rupture was 29.3 months (range 1–98 months). Two patients underwent endovascular relining of their grafts, both of whom survived and were successfully discharged. Two other patients underwent open surgical repair, one of whom survived, the second died due to multi-organ failure a few weeks after the surgery. Three patients were palliated following aneurysm sac rupture due to frailty and subsequently died.

There were 7 procedure-related complications (7%). Non-target embolisation occurred in five cases—in one patient, non-target embolisation to several lumbar branches resulted in vertebral body collapse at L4 and subsequent osteomyelitis. This was managed conservatively with no permanent sequelae, and two others had self-limiting back pain. One arterial injury to the middle colic artery required embolisation and another developed retroperitoneal haematoma during DSP access.

Overall, 27 patients died during follow-up, four of whom were directly related to aneurysm sac rupture as described above. There were two deaths which were suspected but not confirmed to be due to sac rupture in patients with known endoleaks, who died at their local centres. One included a patient with a T2EL with sac expansion confirmed on CT awaiting embolisation. The other was a patient who had a suspected T3a endoleak who died locally prior to further intervention. The remaining 21 deaths were unrelated.

### Comparison of Techniques

The techniques and embolic agents used are summarised in Table 3. The most common approach was transarterial (70/100), with 4/100 transarterial perigraft approaches performed. DSP was the second most common (24/100) with a significant increase in the number of DSPs being performed in our cohort over time—2 undertaken in the first 9 years compared with 22 in the second 9 years.

**Table 3:** Technique and embolic agents used

	**Number (%)**
Technique:	
Transarterial	68 (68)
Transiliac perigraft	6 (6)
Direct sac puncture	24 (24)
Other – transcaval, direct gluteal artery puncture	2 (2)
Embolic agent:	
Liquid	41 (52)
* EVOH*	*29*
* NBCA*	*9*
* PHIL (Precipitating Hydrophobic Injectable Liquid)*	*3*
Liquid + coils/ plug	19 (24)
* EVOH + coils/ plug*	12
* NBCA + coils/ plug*	7
Coils/ plugs alone	13 (16)
Coils and particles	4 (5)
Particles alone	2 (3)

When comparing outcomes between these techniques (Table 4), DSP embolisation was found to be superior to transarterial embolisation in achieving higher rates of technical success and permitting embolisation of the endoleak cavity/nidus (*X*^2^ *p*-value=0.018, OR=8.52 [95%CI 1.27-92.26]). However, no statistical correlation could be found between clinical success and DSP.

**Table 4:** Technical and clinical success rates comparing techniques

	Technical success - number (%)	Chi-square pvalue	Odds ratio (95% CI)	Clinical success – number (%)	Chi-square *p*-value	Odds ratio (95% CI)
DSP	23 (95.8)			17 (70.8)		
Transarterial	50 (73.5)	**0.02**	**8.28 (1.42 – 90.1)**	46 (67.6)	0.77	1.16 (0.43 – 2.99)
Nidus embolised	55 (100)			39 (70.9)		
Nidus not embolised	22 (88)	**0.009**	**17.27 (2.01 – >100)**	16 (66.7)	0.706	1.22 (0.41 – 3.54)
Liquid embolic used	59 (98.3)			41 (73.2)		
Liquid embolic not used	18 (90)	0.089	6.56 (0.71 – 96.2)	11 (55)	0.133	2.24 (0.76 – 6.31)

Embolisation of the endoleak nidus was achieved in 55% of procedures and resulted in greater technical success (*X*^2^ *p*-value=0.009, OR=17.27 [95%CI 2.01– >100]). However, there was no statistically significant correlation with the clinical success rate.

Liquid embolics were used most frequently, followed by a combination of liquid and mechanical embolics (coils or vascular plugs). The use of liquid embolics approached but did not reach statistical significance with technical success (*X*^2^ *p*-value=0.089, OR=6.56 [95%CI 0.71-96.2]). Similarly, the choice of embolic material did not significantly impact clinical success.

## Discussion

We demonstrated 77% technical success and 67% clinical success following embolisation. A systematic review of 59 studies with a cumulative cohort of 1073 patients performed by Ultee et al. [20], identified technical success rates of 87.9% and a stable/reduced aneurysm sac size in 78.4%.

Most of our technical failures were transarterial approaches of LA T2ELs, which are inherently more challenging and associated with poorer success compared to IMA T2ELs [1, 16]. Furthermore, our preference was to re-schedule patients for embolisation via DSP in the event of failure from a transarterial approach. Thirteen patients were rescheduled for this reason and the secondary rate of technical success per patient was higher at 86.1%, comparable to 88.1% in the literature [20]. Additionally, Ultee et al. reported that only 373/1083 cases had documented follow-up aneurysm sac diameters at 12 months post-procedure, which fell to 157/1073 cases at 24 months. We suggest that our longer follow-up (mean—28.6 months) may be a contributing factor for our slightly lower late clinical success rates.

Our results (Table 4) support the current consensus that embolisation of the endoleak nidus and DSP are associated with improved technical success [1, 6, 16]. A proposed mechanism is that DSP allows direct embolisation of EL nidus and inflow/outflow vessels [21, 22]. However, DSP is often reserved for cases where transarterial embolisation has been unsuccessful or the anatomy for a transarterial approach is unfavourable, leaving the proposed superiority of DSP confounded [16, 21]. Additionally, whilst these factors were associated with improved rates of technical success, we did not identify a significant correlation with clinical success. This may be influenced by the variable length of follow-up and the occurrence of new endoleaks.

Exclusive use of coils/vascular plugs are associated with suboptimal outcomes and often require repeat embolisation [1, 23]. Some studies have demonstrated superior results with liquid embolics [23, 24]. Whilst our findings appear to follow this trend, we were unable to identify any statistical significance comparing the use of liquid embolics and other embolic agents in achieving technical success. This may be due to the relatively low number of embolisations performed without a liquid embolic (16 compared to 60 in which a liquid embolic was utilised).

Embolisation of T2ELs has a favourable safety profile with complications usually arising from non-target embolisation of vital structures [1, 20]. This was mirrored in our cohort and most complications were CIRSE grade 1 and 2 [25]. One patient developed a grade 3 complication due to non-target embolisation of EVOH resulting in L4 vertebral body collapse and osteomyelitis. This was managed conservatively with long-term antibiotics without any permanent neurological sequalae.

Noteworthy findings were the endoleak status and rupture rate. Whilst the clinical success rates were 67%, 34 cases had persistent or partially treated T2ELs on surveillance. Of these, less than half were associated with sac size increase and just over half (53%) with either stable or reduced sac size. Furthermore, the rupture rate was 7%, higher than reported rates of up to 2% [1, 17]. Only one rupture (1%) was associated with a persistent T2EL. Of the remainder, 1 patient did not have an endoleak identified on surveillance and 4 were associated with new T1 or T3ELs. This highlights the need for ongoing surveillance despite an apparently adequately treated T2EL.

A propensity-matched analysis on a registry of 4957 patients performed by a Japanese group [26] reported statistically significant rates of aneurysm sac enlargement >5 mm (27.4% versus 2.7%), re-intervention (14.9% versus 0.7%), rupture (0.8% versus 0.1%) and aneurysm-related mortality (1% versus 0.2%) in patients with persistent T2EL compared to those without a T2EL [23]. One reason for this may be that slow growth associated with persistent/secondary T2ELs alters the morphology of the aneurysm sac, potentially predisposing to T1/T3ELs [10–12, 16, 26]. Indeed, 8 patients in our cohort required further endovascular management due to the development of T1 or T3ELs whilst 4/7 cases of rupture had new T1 or T3ELs confirmed on imaging. Overall, our findings support the current consensus to treat T2ELs with sac expansion to prevent changes in aneurysm sac morphology and formation of T1/T3ELs [1, 16].

### Limitations

There are limitations to our study. Firstly, retrospective data collection is associated with selection bias. Secondly, there was variability in the follow-up period, as patients were not under prospective surveillance at defined time points. Third, our sample size was small consisting of 100 procedures in 72 patients. However, our cohort is larger than most reported in the literature, highlighting the lack of data currently available.

Our institution does not routinely perform delayed phase CTs for imaging follow-up, which may underestimate the number of T2ELs. However, patients with clinically significant sac expansion would undergo catheter angiography and a T2EL may be identified and treated at this point despite being occult on CTA. Finally, there was no standardisation in the type of embolic agent utilised. The procedures were performed by experienced vascular interventional radiologists and the embolic choice was dependant on microcatheter position and morphology of the endoleak.

## Conclusion

Our results support the current consensus to embolise T2ELs associated with sac expansion. Endovascular management of T2ELs has a favourable safety profile and embolisation of the endoleak nidus has greater rates of technical success. However, 30% may still exhibit sac growth at 24 months follow-up, confirming the need for ongoing surveillance which should probably be lifelong. There are currently no high-quality data to recommend the optimal access route for embolisation or which specific embolic agent should be used. Each embolisation procedure should be tailored to individual patients depending on their endoleak anatomy.
